# An altered transcriptome underlies *cln5*-deficiency phenotypes in *Dictyostelium discoideum*

**DOI:** 10.3389/fgene.2022.1045738

**Published:** 2022-11-10

**Authors:** William D. Kim, Robert J. Huber

**Affiliations:** ^1^ Environmental and Life Sciences Graduate Program, Trent University, Peterborough, ON, Canada; ^2^ Department of Biology, Trent University, Peterborough, ON, Canada

**Keywords:** Batten disease, CLN5, *Dictyostelium discoideum*, enzyme activity, neuronal ceroid lipofucinosis, proteasome, RNA-seq

## Abstract

Mutations in *CLN5* cause a subtype of neuronal ceroid lipofuscinosis (NCL) called CLN5 disease. The NCLs, commonly referred to as Batten disease, are a family of neurodegenerative lysosomal storage diseases that affect all ages and ethnicities globally. Previous research showed that CLN5 participates in a variety of cellular processes. However, the precise function of CLN5 in the cell and the pathway(s) regulating its function are not well understood. In the model organism *Dictyostelium discoideum*, loss of the *CLN5* homolog, *cln5*, impacts various cellular and developmental processes including cell proliferation, cytokinesis, aggregation, cell adhesion, and terminal differentiation. In this study, we used comparative transcriptomics to identify differentially expressed genes underlying *cln5*-deficiency phenotypes during growth and the early stages of multicellular development. During growth, genes associated with protein ubiquitination/deubiquitination, cell cycle progression, and proteasomal degradation were affected, while genes linked to protein and carbohydrate catabolism were affected during early development. We followed up this analysis by showing that loss of *cln5* alters the intracellular and extracellular amounts of proliferation repressors during growth and increases the extracellular amount of conditioned medium factor, which regulates cAMP signalling during the early stages of development. Additionally, *cln5* ^*-*^ cells displayed increased intracellular and extracellular amounts of discoidin, which is involved in cell-substrate adhesion and migration. Previous work in mammalian models reported altered lysosomal enzyme activity due to mutation or loss of *CLN5*. Here, we detected altered intracellular activities of various carbohydrate enzymes and cathepsins during *cln5* ^*-*^ growth and starvation. Notably, *cln5* ^*-*^ cells displayed reduced β-hexosaminidase activity, which aligns with previous work showing that *D. discoideum* Cln5 and human CLN5 can cleave the substrate acted upon by β-hexosaminidase. Finally, consistent with the differential expression of genes associated with proteasomal degradation in *cln5* ^*-*^ cells, we also observed elevated amounts of a proteasome subunit and reduced proteasome 20S activity during *cln5* ^*-*^ growth and starvation. Overall, this study reveals the impact of *cln5*-deficiency on gene expression in *D. discoideum*, provides insight on the genes and proteins that play a role in regulating Cln5-dependent processes, and sheds light on the molecular mechanisms underlying CLN5 disease.

## 1 Introduction

The neuronal ceroid lipofuscinoses (NCLs), also known as Batten disease, are a family of neurodegenerative diseases linked to mutations in 13 ceroid lipofuscinosis neuronal (*CLN*) genes (*CLN1-8*, *CLN10-14*) (Schulz et al., 2013; Mole and Cotman, 2015). Each of the 13 different subtypes of NCL are characterized by the lysosomal accumulation of autoflourescent lipid-protein aggregates called ceroid lipofuscin in neurons, as well as other cell types outside the central nervous system (Palmer et al., 1992; Mole and Cotman, 2015). The accumulation of ceroid lipofuscin has been associated with numerous clinical symptoms, including seizures, reduced motor, visual, and cognitive function, as well as a reduced lifespan (Schulz et al., 2013; Mole and Cotman, 2015). While most of the *CLN* genes have been studied, the precise cellular mechanisms impacted by *CLN* gene mutations remain elusive.

Mutations in *CLN5* cause the CLN5 disease subtype of NCL (Schulz et a., 2013; Mole and Cotman, 2015). CLN5 is a soluble lysosomal and extracellular protein that is predicted to function as either a glycoside hydrolase or depalmitoylase (Isosomppi et al., 2002; Hughes et al., 2014; Jules et al., 2017; Huber and Mathavarajah, 2018a; Basak et al., 2021b; Luebben et al., 2022). CLN5 has been linked to several cellular processes including, but not limited to, endosomal sorting, biometal homeostasis, sphingolipid metabolism, and autophagy (El Haddad et al., 2012; Mamo et al., 2012; Grubman et al., 2014; Doccini et al., 2020; McLaren et al., 2021). However, like most CLN proteins, the association of CLN5 with a defined biological pathway is still under investigation.

*Dictyostelium discoideum* is a eukaryotic microbe that is used as a biomedical model system for studying a variety of human diseases, including the NCLs (Huber, 2016; Huber et al., 2022). The *D. discoideum* life cycle is comprised of single cell and multicellular phases that allow for a diversity of fundamental cellular and developmental processes to be examined in great biochemical detail (Mathavarajah et al., 2017). During the growth phase of the life cycle, haploid amoebae feed on a food source (in nature: microorganisms in the soil; in the laboratory: bacteria and nutrient-rich liquid media) and divide mitotically. When starved, amoebae initiate a 24-h multicellular developmental programme that begins with the chemotactic aggregation of cells. The multicellular mounds that form then undergo a series of morphological changes to form motile slugs. During the later stages of multicellular development, cells within slugs terminally differentiate to form fruiting bodies composed of stalk cells and viable spores that can restart the life cycle when nutrients become available.

The *D. discoideum* genome encodes homologs for 11 of the 13 human CLN proteins (Huber, 2016; Huber et al., 2020). Our previous work showed that the homolog of human CLN5, Cln5, has glycoside hydrolase activity and is secreted (Huber and Mathavarajah, 2018a). Our work also linked the function of Cln5 in *D. discoideum* to cell proliferation, cytokinesis, folic acid-mediated chemotaxis, and autophagy during growth, and since cAMP-chemotaxis, aggregation, and developmental timing during multicellular development (Huber and Mathavarajah, 2018b; McLaren et al., 2021).

In this study, we used comparative transcriptomics to identify genes that are impacted by *cln5*-deficiency in *D. discoideum* during growth and after 4 h of starvation (when *cln5* expression is maximal) (Stajdohar et al., 2017). During growth, genes associated with protein ubiquitination/deubiquitination, cell cycle progression, and proteasomal degradation were affected, while genes linked to protein and carbohydrate catabolism were affected in cells starved for 4 h. We then showed that loss of *cln5* affects the levels of proliferation repressors during growth and proteins required for cAMP-mediated chemotaxis and adhesion during the early stages of multicellular development. Finally, we showed that deletion of *cln5* affects the activities of lysosomal enzymes and the proteasome. Together, this study examines the impact of *cln5*-deficiency on the *D. discoideum* transcriptome and provides further insight into the multifaceted role of CLN5 in the eukaryotic cell.

## 2 Materials and methods

### 2.1 Cell lines, media, and antibodies

AX3 (hereafter referred to as WT, parental cell line of *cln5* ^*-*^) and *cln5* ^*-*^ cells were maintained on SM/2 agar containing *Klebsiella aerogenes* (Fey et al., 2007). Cells used in experiments were cultured axenically in nutrient-rich medium (HL5) at 22°C and 150 RPM (Formedium, Hunstanton, Norfolk, United Kingdom). HL5 was supplemented with streptomycin sulfate (300 μg/ml) and ampicillin (100 μg/ml) to prevent bacterial growth. Blasticidin S hydrochloride (10 μg/ml) was used to select *cln5* ^*-*^ cells. For all experiments, cells were harvested during the mid-log phase of growth (1–5 x 10^6^ cells/ml). KK2 buffer was formulated as follows: 0.7 g/L K_2_HPO_4_, 2.2 g/L KH_2_PO_4_, pH 6.5. Mouse monoclonal anti-cortexillin-I (CtxA) (241-438-1) (Faix et al., 2001), mouse monoclonal anti-cortexillin-II (CtxB) (232-238-10) (Faix et al., 2001), mouse monoclonal anti-discoidin (Dsc) (80-52-13) (Wetterauer et al., 1993), mouse monoclonal myosin heavy chain type II (MhcA) (56-396-5) (Pagh and Gerisch, 1986), mouse monoclonal anti-proteasomal subunit (PS) (159-183-10) (Schauer et al., 1993), and mouse monoclonal anti-proteasomal subunit 5 (PS5) (171-337) (Schauer et al., 1993) were purchased from the Developmental Studies Hybridoma Bank (University of Iowa, Iowa City, Iowa, United States). Mouse monoclonal anti-β-actin (SC-47778) was purchased from Santa Cruz Biotechnology Incorporated (Dallas, Texas, United States). Rabbit polyclonal anti-autocrine proliferation repressor (AprA) (Brock and Gomer, 2005), rabbit polyclonal anti-counting factor-associated protein D (CfaD) (Bakthavatsalam et al., 2008), and rabbit polyclonal anti-conditioned medium factor (CmfA) (Jain et al., 1992) were generous gifts from Dr. Richard Gomer (Texas A&M University, Texas, United States). HRP-linked secondary antibodies were purchased from New England Biolabs Canada (Whitby, Ontario, Canada).

### 2.2 RNA preparation

**FIGURE 1:**
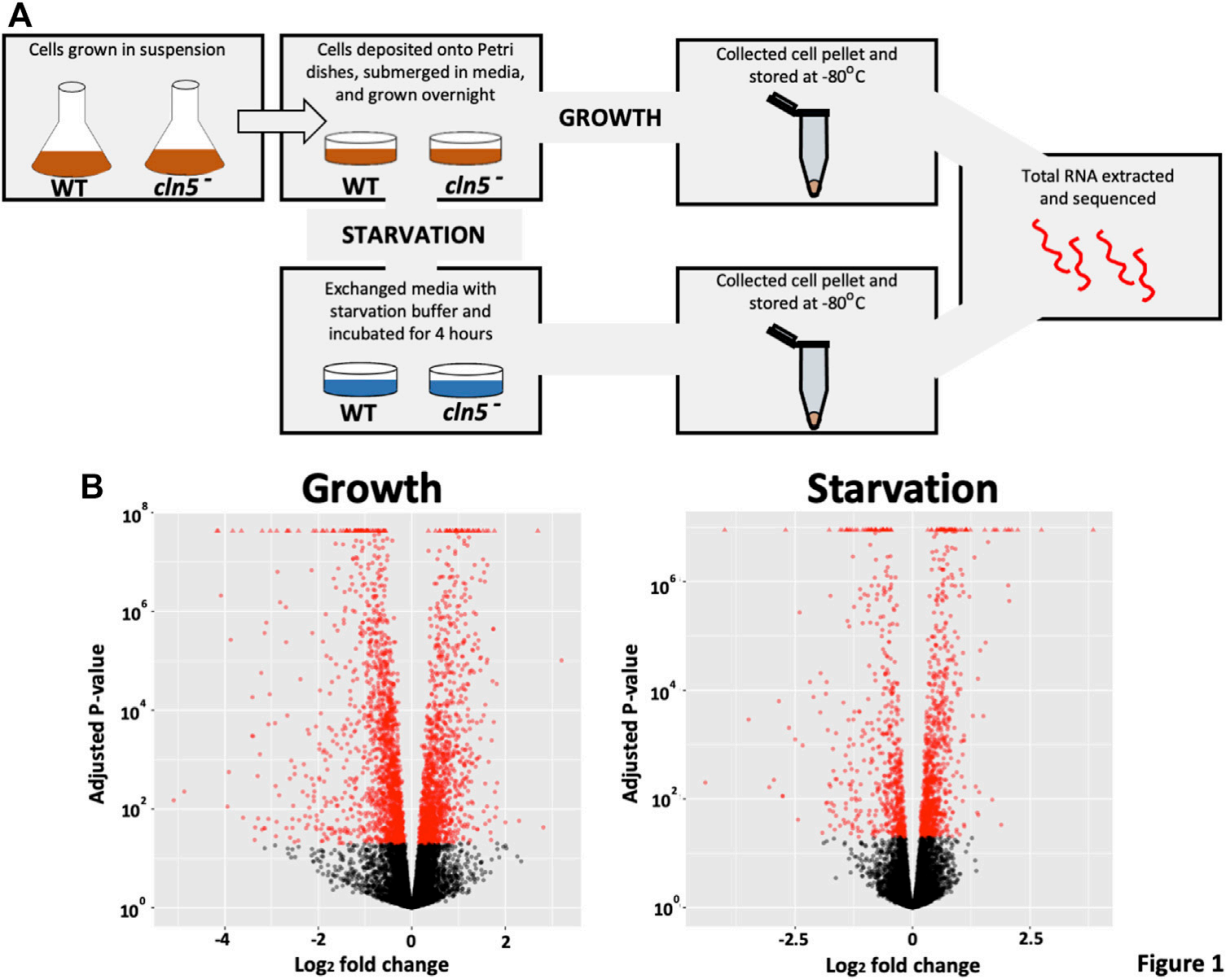
A pictorial schematic of the methods used to determine the effects of *cln5* deletion on gene expression. **(A)** WT and *cln5* ^*-*^ cells grown in suspension and in the mid-log phase of growth were deposited onto Petri dishes and grown overnight in HL5. Growth-phase cells were harvested after the overnight incubation, while starved cells were collected after a 4-hour incubation in KK2 buffer. All cell pellets were stored at −80°C until RNA extraction was done. Extracted RNA samples were sequenced at The Hospital for Sick Children (Toronto, Ontario, Canada). **(B)** Following differential expression analysis *via* SARTools, volcano plots were generated to visualize the profile of DEGs during growth and after 4 h of starvation.

Cells grown in suspension were deposited onto Petri dishes containing HL5 medium and left to adhere to the dish for at least 1 h at 22°C (Figure 1A). The medium was replaced with fresh HL5 medium containing ampicillin (100 μg/ml) and streptomycin sulphate (300 μg/ml) and left overnight to allow for two mitotic doubling times. The following day, growth-phase cells and cells starved for 4 h in KK2 buffer were harvested and stored at −80°C for future use. A 4-h starvation timepoint was selected since this is when *cln5* is maximally expressed in *D. discoideum* (http://dictyexpress.biolab.si/) (Stajdohar et al., 2017). Growth-phase and starved cells from −80°C storage were lysed and RNA was extracted using the Monarch Total RNA Miniprep Kit according to the manufacturer’s instructions (New England Biolabs Canada, Whitby, Ontario, Canada). A total of three biological replicates each for growth and starvation were submitted for RNA sequencing (RNA-seq) analysis.

### 2.3 RNA sequencing and bioinformatics

RNA-seq was performed by The Centre for Applied Genomics at the Hospital for Sick Children (Toronto, Ontario, Canada). All RNA samples were prepared with a stranded poly(A) mRNA library preparation kit (NEBNext, New England Biolabs Canada, Whitby, Ontario, Canada) and the quality of RNA was determined using a Bioanalyzer (Agilent Technologies, Santa Cruz, California, United States). The RNA-seq analysis for all mRNA libraries were done through an Illumina NovaSeq SP flowcell PE100 sequencer (San Diego, California, United States). Adaptor sequences attached to the raw paired reads were removed with Trimmomatic (Bolger et al., 2014). The quality of the raw paired reads from the sequencer was assessed with FastQc (https://www.bioinformatics.babraham.ac.uk/projects/fastqc/). The *D. discoideum* reference genome acquired from the Ensembl database was indexed with hierarchical indexing for spliced alignment of transcripts 2 (HISAT2) and the paired reads were mapped onto the indexed reference genome with HISAT2 (Kim et al., 2019). To obtain read counts, FeatureCounts was used on the mapped reads (Liao et al., 2014). The differential gene expression analysis was done using the DeSeq2 version of statistical analysis of RNA-seq data tools (SARTools) package (Love et al., 2014; Varet et al., 2016). In the SARTools parameters, an alpha value of 0.05 along with the Benjamin-Hochberg (BH) multiple-testing correction were used in the differential gene expression analysis. Furthermore, a log_2_ fold change threshold of 1.25 was applied on the list of differentially expressed genes (DEGs) (see Supplementary Table S1 for full list of DEGs). Logically accelerated gene ontology term finder (LAGO) was used for gene ontology (GO) term enrichment analyses of the DEGs (Boyle et al., 2004). A *p*-value of 0.05, combined with the BH correction was applied. Within each GO term enrichment analysis (e.g., biological process, molecular function, and cellular component), only annotated genes that were identified by the LAGO database were used.

### 2.4 Enzyme activity assays

Growth-phase cells and cells starved for 4 h were lysed with 0.1% NP40 in 0.05 M 2-(N-morpholino) ethanesulfonic acid (MES) (pH 6.5). All enzyme activity assays were performed using equal amounts of protein from whole cell (WC) lysates (100-200 µg). Proteins in WC lysates were quantified using a Qubit 2.0 Fluorometer (Fisher Scientific Company, Ottawa, Ontario, Canada). Each assay was performed in triplicate and measured using a Synergy HTX multi-mode plate reader (BioTek Instruments Incorporated, Winooski, Vermont, United States). All activity values were subtracted from values acquired from the blank solution. Enzyme activities in *cln5* ^*-*^ WC lysates were standardized against activities in WC lysates from WT cells. Data were statistically analyzed using the one-sample *t*-test and a *p*-value < 0.05 was considered significant.

#### 2.4.1 α-mannosidase and β-glucosidase

For α-mannosidase, methods were adapted from Loomis (1970). Briefly, WC lysates were added to 5 mM acetate buffer (pH 5.0) containing a final concentration of 5 mM para-nitrophenyl-α-D-mannopyranoside substrate (N2127, Sigma Aldrich Canada, Oakville, Ontario, Canada). For β-glucosidase, methods were adapted from Coston and Loomis (1969) using a final concentration of 10 mM para-nitrophenyl-β-D-glucopyranoside substrate (487507, Sigma Aldrich Canada, Oakville, Ontario, Canada) in 50 mM acetate buffer (pH 5.0). For both assays, reaction mixtures were incubated at 35°C for 45 min, quenched with an equal volume of 1 M Na_2_CO_3_, and activity was measured at 405 nm absorbance.

#### 2.4.2 α-glucosidase

Methods were adapted from Wimmer et al. (1997). Briefly, WC lysates were added to 0.1 M sodium succinate (pH 6.0) containing 2 mM p-nitrophenyl-α-D-glucopyranoside substrate (487506, Sigma Aldrich Canada, Oakville, Ontario, Canada). Reactions were incubated at 65°C for 1 h and then quenched with two equal volumes of 1 M Na_2_CO_3_. Activity was measured at 395 nm absorbance.

#### 2.4.3 α- and β-galactosidase

Methods were adapted from Kilpatrick and Stirling (1976) and Maruhn (1976). The α-galactosidase activity assay was performed using 2 mM para-nitrophenyl α-D-galactopyranoside substrate (N0877, Sigma Aldrich Canada, Oakville, Ontario, Canada) in citrate/phosphate buffer (pH 4.5), while the β-galactosidase activity assay contained a reaction solution of 5 mM ortho-nitrophenyl-β-D-galactopyranoside substrate (48712-M, Sigma Aldrich Canada, Oakville, Ontario, Canada) in 100 mM citrate buffer (pH 4.0). In both assays, the reaction solutions were incubated at 37°C for 45 min. The α-galactosidase reactions were quenched with an equal volume of 1 M sodium glycinate (pH 10.4). β-galactosidase reactions were terminated using an equal volume of 2-amino-2-methyl-propanol/HCl buffer. Activity was measured at 405 nm absorbance.

#### 2.4.4 Palmitoyl-protein thioesterase 1

Methods were modified from van Diggelen et al. (1999). Briefly, WC lysates were added to 0.2 mM 4-methylumbelliferyl 6-thio-palmitate-β-D-glucopyranoside substrate (19524, Cedarlane Labs, Burlington, Ontario, Canada) in McIlvain’s phosphate/citrate buffer containing 15 mM dithiothreitol and 0.375% (v/v) Triton X-100 (pH 4.0). Reactions were incubated for 1 h at 37°C and then stopped by heating the solution for 2 min at 95°C. 1 U of β-glucosidase enzyme (from almonds, dissolved in distilled water containing 0.2% (w/v) bovine serum albumin) (Sigma Aldrich Canada, Oakville, Ontario, Canada) was added to the cooled reaction mixture while adjusting the pH to 5 with NaOH. The samples were left at 37°C for 1 h and then an equal volume of 0.5 M Na_2_CO_3_/NaHCO_3_ buffer containing 0.025% (v/v) Triton X-100 (pH 10.7) was added to terminate the reaction. Fluorescence was detected using the following filters: 360/40 nm excitation, 460/40 nm emission.

#### 2.4.5 Tripeptidyl peptidase 1

Methods were adapted from Stumpf et al. (2017). Briefly, WC lysates were combined with 120 µM Ala-Ala-Phe-7-amido-4-methylcoumarin tripeptidyl peptidase 1 (TPP1) substrate (A3401, Sigma Aldrich Canada, Oakville, Ontario, Canada) in buffer containing 100 mM sodium acetate/150 mM NaCl/0.1% (v/v) Triton X-100 (pH 4.5). Reactions were incubated at 37°C for 1 h in the dark, after which time an equal volume of quenching solution (100 mM sodium acetate, 150 mM NaCl, pH 4.3) was added to stop the reaction. Fluorescence was detected using the following filters: 360/40 nm excitation, 460/40 nm emission.

#### 2.4.6 Cathepsin D and cathepsin F

Cathepsin D (CTSD) enzymatic activity was assessed using the CTSD Activity Assay Kit according to the manufacturer’s instructions (10013-596, VWR International, Mississauga, Ontario, Canada) and fluorescence was detected using the following filters: 360/40 nm excitation, 460/40 nm emission. Cathepsin F (CTSF) activity was measured using a method adapted from Fonovič et al. (2004). Briefly, WC lysates were mixed with 5 μg/ml pepsin (10108057001, Sigma Aldrich Canada, Oakville, Ontario, Canada) (pH 4.5–5.0). Reactions were preincubated for 1 h at 37°C, after which time 1 µM Z-FR-AMC substrate (80350BP, Cedarlane Labs, Burlington, Ontario, Canada), as well as 0.1 M sodium phosphate buffer containing 1 mM EDTA and 0.1% (v/v) PEG 600 (pH 6.5) were added to the reaction mixture. The reaction was adjusted to a final DMSO content of 5% (v/v) and then incubated at 27°C for 1 h. Fluorescence was detected using the following filters: 360/40 nm excitation, 460/40 nm emission.

#### 2.4.7 Cathepsin B

Methods were adapted from Barrett (1980). Briefly, samples were mixed with reaction buffer (352 mM KH_2_PO_4_, 48 mM Na_2_HPO_4_, 4 mM disodium EDTA) supplemented with fresh cysteine at a final concentration of 8 mM. Reactions were preincubated for 5 min at 40°C, after which time they were incubated for 1 h at 40°C in the presence of 1.5 mM of fluorogenic cathepsin B (CTSB) substrate (219392, Sigma Aldrich Canada, Oakville, Ontario, Canada). Reactions were stopped with two equal volumes of 100 mM sodium chloroacetate/30 mM sodium acetate/70 mM acetic acid buffer (pH 4.3) and fluorescence was detected using the following filters: 360/40 nm excitation, 460/40 nm emission.

#### 2.4.8 N-acetylglucosaminidase

Methods were adapted from Loomis (1969) and Huber and Mathavarajah (2018a). Briefly, WC lysates were mixed with 4.2 mM para-nitrophenyl N-acetyl-β-D-glucosaminide substrate (N9376, Sigma Aldrich Canada, Oakville, Ontario, Canada) in 100 mM acetate buffer (pH 5.0). Reactions were incubated for 5 min at 35°C. An equal volume of 1 M Na_2_CO_3_ was added to stop the reaction. Activity was measured at 405 nm absorbance.

### 2.5 Proteasome activity

Proteasomal activity was assessed using the Proteasome 20S Activity Assay Kit according to the manufacturer’s instructions (MAK172, Sigma Aldrich Canada, Oakville, Ontario, Canada). Briefly, growth-phase cells and cells starved for 4 h were lysed with 0.1% (v/v) NP40 in 0.05 M MES buffer (pH 6.5) and equal protein amounts (100-150 µg) from WC lysates were used in the assay. Proteins in WC lysates were quantified using a Qubit 2.0 Fluorometer. Samples were incubated for 1 h at 37°C and activity was measured using a Synergy HTX multi-mode plate reader and the following filters: 360/40 nm excitation, 460/40 nm emission. All activity values were subtracted from the blank solution and reads obtained from the c*ln5* ^*-*^ line were normalized to WT activity values. Data were statistically analyzed using the one-sample *t*-test and a *p*-value of <0.05 was considered significant.

### 2.6 SDS-PAGE and western blotting

WC lysates were prepared from growth-phase cells and cells starved for 4 h. In addition, WT and *cln5* ^*-*^ conditioned media (CM) was collected during growth and conditioned buffer (CB) was collected after 4 h of starvation. CM and CB were clarified through centrifugation (4°C/1500 RPM/5 min). Protein concentrations were determined using a Qubit 2.0 Fluorometer. SDS-PAGE and western blotting were performed using standard methods (2-hour incubation at 22°C for primary and secondary antibodies in 5% (w/v) milk/TBST. The following antibody dilutions were used: anti-AprA (1:1,000), anti-CfaD (1:1,000), anti-CtxA (1:2,000), anti-CtxB (1:2,000), anti-MhcA (1:1,000), anti-CmfA (1:1,000). anti-Dsc (1:1,000), anti-PS (1:1,000), anti-PS5 (1:1,000), and anti-β-Actin (1:1,000). All primary antibodies used for western blotting detected proteins at molecular weights that were consistent with previous studies (see Section 2.1 for citations). HRP-linked secondary antibodies were used at a dilution of 1:2,000. The ChemiDoc Imaging System (Bio-Rad Laboratories Canada, Mississauga, Ontario, Canada) was used to digitally scan the immunoblots. Protein bands were quantified using Fiji/ImageJ and values obtained from WC lysates were normalized to the corresponding levels of β-Actin (Schindelin et al., 2012). Values obtained from *cln5* ^*-*^ samples were standardized against WT values and the one-sample *t*-test was used to assess statistical significance. A *p*-value of <0.05 was considered significant.

## 3 Results

### 3.1 Comparative transcriptomics reveals the effects of *cln5*-deficiency on gene expression in *D. discoideum*

Previous work in *D. discoideum* linked the function of Cln5 to cell proliferation, cytokinesis, folic acid-mediated chemotaxis, and autophagy during growth, and cyclic adenosine monophosphate (cAMP)-mediated chemotaxis, adhesion, and aggregation during the early stages of multicellular development (Huber and Mathavarajah, 2018b; McLaren et al., 2021). To gain insight into the molecular mechanisms underlying *cln5*-deficiency phenotypes in *D. discoideum* and the biological pathways impacted by the loss of *cln5*, we performed RNA-seq analysis on growth-phase cells and cells starved for 4 h in KK2 buffer. We chose the 4-hour timepoint since this is when *cln5* expression is maximal (Stajdohar et al., 2017). The list of DEGs was filtered to include only those genes with a log_2_ fold change of 1.25 (increase or decrease) and a *p*-value < 0.05. A volcano plot was generated to visualize the profile of DEGs (Figure 1B). RNA-seq revealed 2324 unique DEGs in *cln5* ^*-*^ cells during growth that included 1041 upregulated genes and 1283 downregulated genes (including *cln5*) (Table 1). After 4 h of starvation, 644 genes were upregulated in *cln5* ^*-*^ cells and 394 were downregulated (including *cln5*), totalling 1038 unique genes.

**TABLE 1:** Quantitative summary of DEGs and annotated genes determined by LAGO in *cln5* ^*-*^ cells during growth and after 4 h of starvation. A log_2_ fold threshold of 1.25 was applied.

				LAGO annotated genes		
Condition	Number of up-regulated genes	Number of down-regulated genes	Total DEGs	Biological process	Cellular component	Molecular function
Growth	1,041	1,283	2,324	1,603	1,652	1,586
Starvation	644	394	1,038	657	728	669

During growth, the most significantly downregulated genes were those encoding heat shock proteins (*hspE-1, hspG3*, *hspG4, hspG5, hspG6, hspG7, hspG8, hspG12, hspH, hspJ, hspM, dnaja1*) and genes involved in protein ubiquitination (*ubqG, ubqH, ubqI, ubqJ, DDB_G0285907*) (Supplementary Table S1). Conversely, the most significantly upregulated genes were those associated with cell cycle progression, including but not limited to, subunits of the anaphase-promoting complex (*anapc3, anapc5, anapc6, anapc7, anapc10*), and genes involved in mitosis, such as kinesin-related genes (*kif2, kif4, kif10, kif12, kif13*) (Castro et al., 2005; Nag et al., 2008; Tikhonenko et al., 2009; Koonce, 2020). During starvation, genes related to development (*cotC, cotD, cotE*) were significantly downregulated in *cln5* ^*-*^ cells. In contrast, genes encoding ADP-ribosylation factors (*arrJ, arrH, arrK*), which modulate the trafficking of endocytic and secretory vesicles, and ponticulin-like proteins (*ponC1, ponC2, ponC3, ponC4, ponC5*), which are involved in actin bundling and cell-cell adhesion, were the most significantly upregulated genes in *cln5*-deficient cells (Ingalls et al., 1989; Shariff and Luna, 1990; Chia et al., 1993; Li and Guo, 2022).

### 3.2 GO term enrichment analyses of differentially expressed genes in *cln5* ^*-*^ cells

As a first step towards analyzing DEGs during growth and starvation, we performed GO term enrichment analyses using LAGO; an online program that clusters genes based on common biological processes they have been associated with, molecular functions of the proteins encoded by the genes, and the subcellular localizations of the proteins (Boyle et al., 2004). For this analysis, we excluded those genes that were uncharacterized (e.g., annotated, but unknown function) (Table 1). Out of 2324 DEGs during growth, this resulted in a list of 1603 genes for biological process, 1586 genes for molecular function, and 1652 genes for cellular component. During growth, DEGs associated with *cln5*-deficiency are linked to a variety of biological processes including macroautophagy (20 genes), protein ubiquitination (60 genes) and deubiquitination (15 genes), proteasomal-mediated ubiquitin-dependent protein catabolism (56 genes), signal transduction (147 genes), and lipid metabolism (111 genes) (Supplementary Table S2). Proteins encoded by DEGs during growth have kinase activity (75 genes), hydrolase activity (330 genes), and bind to a variety of biological substrates (e.g., protein, 228 genes; carbohydrates, 296 genes; lipids, 44 genes; nucleotides, 323 genes; ions, 535 genes). In addition, the protein products of the DEGs primarily localize to the nucleus (446 genes), ubiquitin ligase complex (37 genes), cell periphery (171 genes), plasma membrane (139 genes), cytoplasm (672 genes), and cellular components associated with cell division (e.g., microtubule organizing centre, 29 genes; spindle pole, 11 genes; spindle, 20 genes; microtubule cytoskeleton, 39 genes; kinetochore, 10 genes; centromeric region of the chromosome, 15 genes; chromosome, 73 genes).

Like growth, only characterized genes (e.g., annotated with a known or predicted function) from the starvation DEG list were used in LAGO analyses, which included 657, 669, and 728 genes for biological process, molecular function, and cellular component, respectively (Table 1). DEGs during starvation are involved in several biological processes including signal transduction (83 genes), protein phosphorylation (42 genes), and catabolic processes involving carbohydrates (8 genes) and proteins (51 genes) (Supplementary Table S2). Proteins encoded by the DEGs during starvation have a variety of molecular functions including kinase (37 genes) and hydrolase (125 genes) activity and bind to a variety of substrates (carbohydrates, 113 genes; ions, 221 genes; nucleotides, 123 genes; proteins, 100 genes). Additionally, some GO terms related to molecular function were only observed in DEGs during starvation including ubiquitin conjugation (8 genes), cysteine-type endopeptidase activity (14 genes), and actin binding (19 genes). Finally, proteins encoded by DEGs during starvation are associated with lysosomes (25 genes), secretory vesicles (8 genes), the cell periphery (118 genes), the cytoplasm (320 genes), the plasma membrane (78 genes), and extracellularly (20 genes). Overall, LAGO analyses revealed that loss of *cln5* influences the expression of hydrolases and genes involved in protein processing during growth and starvation. In addition, LAGO analyses revealed clusters of DEGs involved in lipid metabolism during growth and carbohydrate metabolism during starvation. Lastly, *cln5*-deficiency appears to impact the expression of genes that encode proteins within the secretory pathway during starvation.

### 3.3 Differentially expressed genes associated with *cln5*-deficiency phenotypes during growth

**FIGURE 2:**
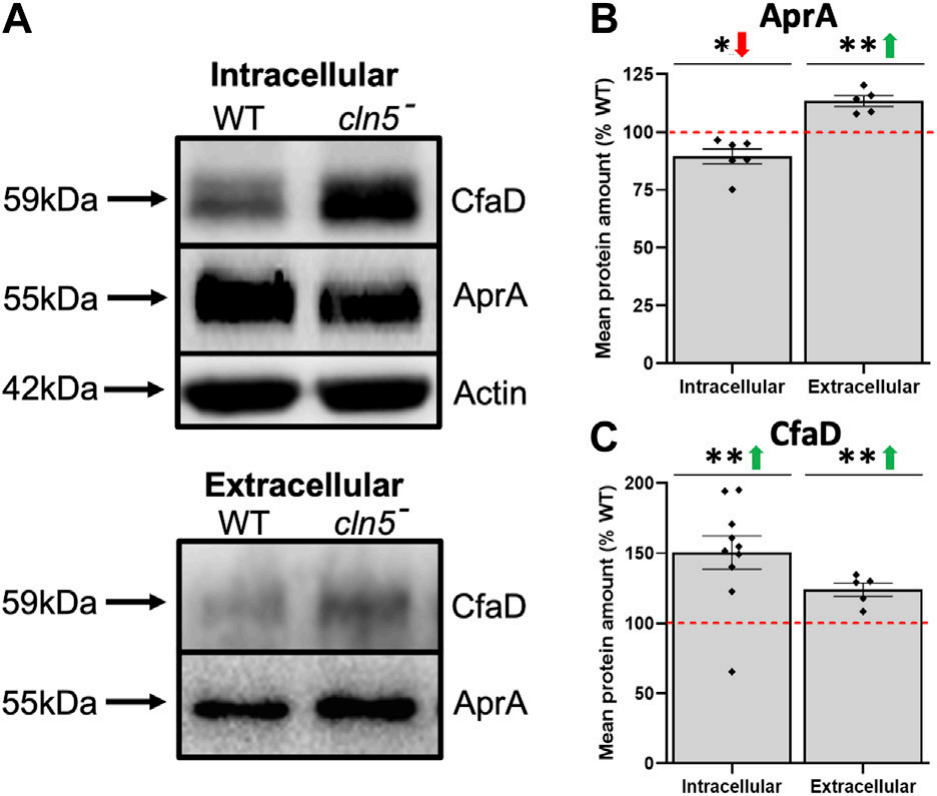
Loss of *cln5* affects the intracellular and extracellular levels of AprA and CfaD during growth. Whole cell (WC) lysates (20-30 µg) and samples of conditioned media (CM) (0.1–0.375 µg) from growth-phase WT and *cln5* ^*-*^ cells were separated by SDS-PAGE and analyzed by western blotting. **(A)** Membranes were probed with anti-AprA, anti-CfaD, and anti-β-actin (loading control). Intracellular and extracellular amounts of **(B)** AprA and **(C)** CfaD were quantified using Fiji/ImageJ and intracellular values were standardized against the levels of β-actin. Data presented as mean protein amount (% WT) ± SEM (*n* ≥ 5). *p < 0.05 (one sample *t*-test).

In *D. discoideum*, *cln5-*deficiency reduces cell proliferation and impairs cytokinesis during growth (McLaren et al., 2021). Here, RNA-seq revealed that loss of *cln5* affects the expression of several genes that encode proteins related to cell proliferation including, but not limited to, kinases (*aurK* and *cdk1,* increased; *qkgA-1*, LRRK family protein kinase, decreased), and proteins involved in cell division (*rblA*, *cdc45*, and *cycB*) (all increased) (Supplementary Table S3) (Luo et al., 1995; Sharma et al., 1999; Li et al., 2008; Phillips and Gomer, 2010; Sanchez-Pulido and Ponting, 2011; Strasser et al., 2012). Loss of *cln5* also increased the expression of *aprA*, which encodes the secreted AprA (Brock and Gomer, 2005; Choe et al., 2009). At the protein level, we observed a decreased amount of intracellular AprA and an elevated amount extracellularly suggesting that loss of *cln5* increases the secretion of AprA (Figures 2A,B). AprA exerts its effect by interacting with CfaD (Bakthavatsalam et al., 2008; Choe et al., 2009). Although *cfaD* was not identified as a DEG during growth, loss of *cln5* increased the intracellular and extracellular amounts of CfaD (Figures 2A,C). Together, these data suggest that Cln5 regulates the levels of AprA and CfaD to modulate cellular proliferation.

**FIGURE 3:**
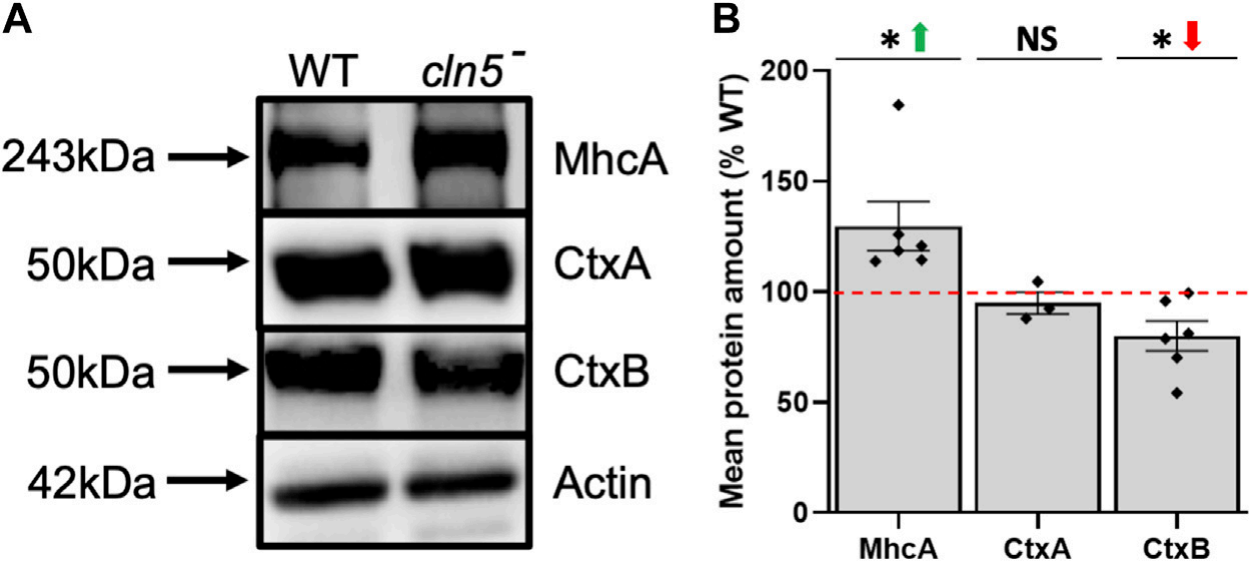
Loss of *cln5* affects the abundance of proteins linked to cytokinesis during growth. **(A)** Whole cell (WC) lysates (20-30 µg) from growth-phase WT and *cln5*-deficient cells were separated by SDS-PAGE and analyzed by western blotting. Membranes were probed with anti-CtxA, anti-CtxB, anti-MhcA, and anti-β-actin (loading control). **(B)** Intracellular amounts of CtxA, CtxB, and MhcA were quantified using Fiji/ImageJ and standardized against the levels of β-actin. Data presented as mean protein amount (% WT) ± SEM (*n* ≥ 3). *p < 0.05 (one sample *t*-test). NS – not significant.

Consistent with the effect of *cln5*-deficiency on cytokinesis, we detected several DEGs during growth that are associated with cytokinesis including vinculin A (*vinA*)*, ctxA,* and *mhcA*, which were all decreased (Supplementary Table S4) (De Lozanne and Spudich, 1987; Faix et al., 1996; Stock et al., 1999; Weber et al., 1999; Nagasaki et al., 2009). In *D. discoideum*, cytokinesis can occur *via* two processes, one is dependent on myosin II and the other on cell adhesion (Zang et al., 1997). CtxA and MhcA participate in myosin II-dependent cytokinesis, while VinA localizes to focal adhesion regions and is thought, along with CtxB, to be involved in cell adhesion-dependent cytokinesis (Zang et al., 1997; Bukharova et al., 2005; Duran et al., 2009; Nagasaki et al., 2009). In addition, cortexillins function as actin-bundling proteins to help form the cleavage furrow during cytokinesis (Weber et al., 1999). To expand upon these findings, we assessed the amounts of CtxA, CtxB, and MhcA protein in *cln5* ^*-*^ cells. Loss of *cln5* had no effect on the amount of CtxA but did decrease and increase the intracellular amounts of CtxB and MhcA, respectively (Figures 3A,B). Overall, these results support the role of Cln5 in cell proliferation and cytokinesis and provide insight into the genes and proteins affected by *cln5*-deficiency during growth.

### 3.4 Differentially expressed genes associated with *cln5*-deficiency phenotypes during starvation

**FIGURE 4:**
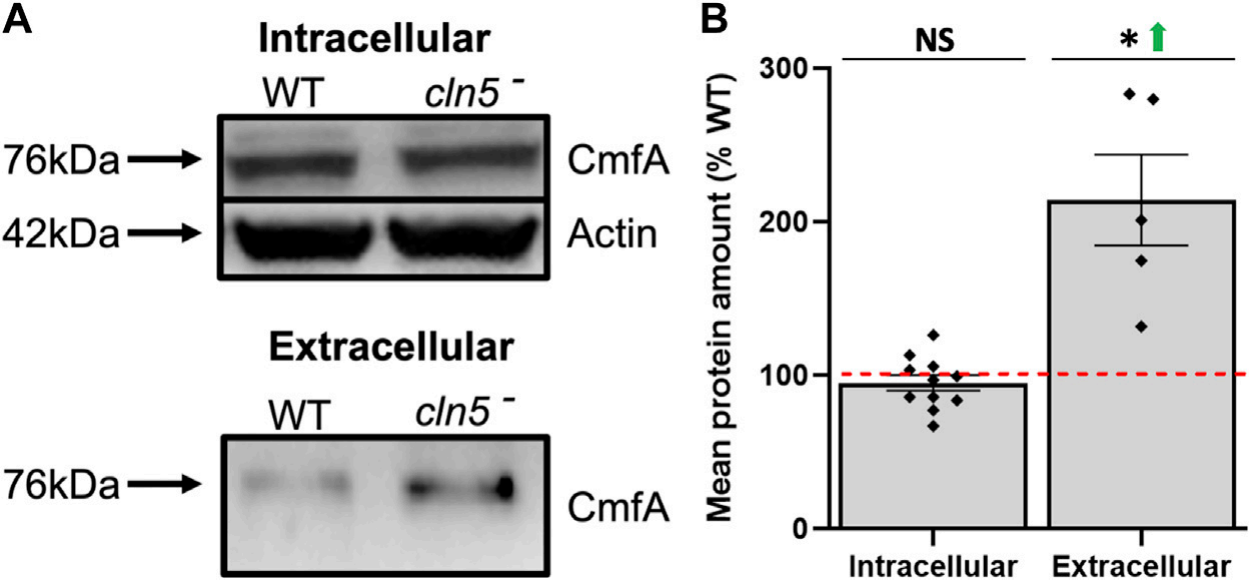
Loss of *cln5* affects the extracellular amount of CmfA during starvation. **(A)** WT and *cln5*-deficient cells starved for 4 h were lysed and standard SDS-PAGE/western blotting was performed on equal protein amounts from whole cell (WC) lysates (20-40 µg) and clarified conditioned buffer (CB) (0.15–0.375 µg). Membranes were probed with anti-CmfA and anti-β-actin (loading control). **(B)** Intracellular and extracellular amounts of CmfA were quantified with Fiji/ImageJ and intracellular amounts of CmfA were standardized against the levels of β-actin. Data presented as mean protein amount (% WT) ± SEM (*n* ≥ 5). *p < 0.05 (one sample *t*-test). NS – not significant.

*cln5*-deficiency in *D. discoideum* delays aggregation, inhibits cAMP-mediated chemotaxis, and reduces adhesion during the early stages of development (Huber and Mathavarajah, 2018b; McLaren et al., 2021). Therefore, we surveyed our list of DEGs during starvation to identify dysregulated genes that may contribute to these phenotypes. When we scanned the starved DEG list for genes that have been linked to delayed aggregation*, myoK* (myosin-K heavy chain) and *rps4* (40S ribosomal protein S4) were increased in expression, while *qkgA-1* was reduced (Supplementary Table S5). RNA-seq also revealed genes that encode proteins involved in cAMP signalling (e.g., cAMP receptor D, *carD*, increased; cAMP-like receptor 4, *crlD,* decreased) and degradation (e.g., 3′,5′-cyclic-nucleotide phosphodiesterase, *regA,* increased) (Supplementary Table S6). The *D. discoideum* genome encodes four receptors that bind cAMP (CarA, CarB, CarC, and CarD) (Kim et al., 1996). *carD* is expressed the highest during the later stages of multicellular development, while cAMP receptor A (*carA*) is expressed the highest during aggregation. RNA-seq revealed that *carA* was not differentially expressed in *cln5* ^*-*^ cells. CarA elevates cAMP synthesis during aggregation by stimulating adenylyl cyclase activity and its expression *via* protein kinase A (Mann et al., 1997; Loomis, 1998; Loomis, 2014). RegA reduces intracellular cAMP levels and leads to reducing cAMP sensitivity in *D. discoideum* (Sun and Devreotes, 1991; Schulkes and Schaap, 1995; Loomis, 1998; Shaulsky et al., 1998; Loomis, 2014). Additionally, cAMP synthesis and binding, as well as the activation of both CarA and early developmental genes, are modulated by CmfA, which binds to the conditioned media factor receptor (CmfB) (Gomer et al., 1991; Yuen et al., 1995; van Haastert et al., 1996; Deery and Gomer, 1999). In our RNA-seq analysis, *cmfA* was not differentially expressed, but *cmfB* was elevated. Furthermore, while loss of *cln5* had no effect on the amount of intracellular CmfA, *cln5*-deficiency elevated the amount of extracellular CmfA (Figures 4A,B). Together, these findings show that *cln5*-deficiency impacts the expression of genes associated with cAMP signalling during aggregation.

**FIGURE 5:**
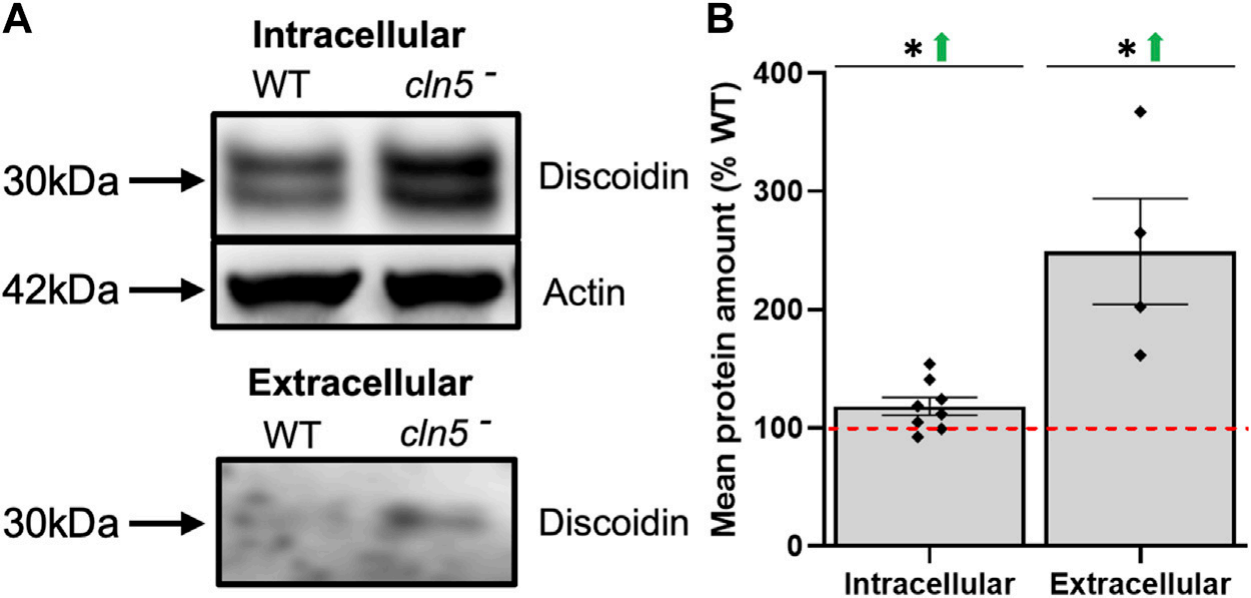
Loss of *cln5* affects the intracellular and extracellular amounts of discoidin during starvation. **(A)** Whole cell (WC) lysates (30-50 µg) and clarified conditioned buffer (CB) (0.15–0.375 µg) were collected from WT and *cln5*-cells starved for 4 h. Equal protein amounts were subjected to SDS-PAGE/western blotting, and membranes were probed with anti-Dsc and anti-β-actin (loading control). **(B)** Protein bands were quantified using Fiji/ImageJ and the intracellular values for discoidin were standardized against the levels of β-actin. Data presented as mean protein amount (% WT) ± SEM (*n* ≥ 4). *p < 0.05 (one sample *t*-test). NS – not significant.

Cell-substrate and cell-cell adhesion also play roles in *D. discoideum* aggregation, which is delayed by the loss of *cln5* (Huber and Mathavarajah, 2018b). In addition to cAMP-mediated chemotaxis, CmfA also influences the expression of discoidin I, a lectin that is secreted and involved in cell-substrate adhesion and migration (Springer et al., 1984; Barondes et al., 1985; Gomer et al., 1991). In scanning the list of DEGs during starvation, genes encoding discoidin subunits (*dscB, dscC, dscD*) were elevated in expression (Supplementary Table S7). In support of the differential expression, elevated amounts of intracellular and extracellular discoidin were observed with the loss of *cln5* (Figures 5A,B). Similarly, *D. discoideum* contains a protein complex composed of counting factors (CF45-1, CF50-1, and CF60) and countin (CtnA), that collectively regulate cell adhesion and migration (Brock and Gomer, 1999; Roisin-Bouffay et al., 2000; Tang et al., 2002). Our RNA-seq analysis revealed elevated expression of *cf45-1* and *cf50-1* in *cln5* ^*-*^ cells during starvation. Furthermore, RNA-seq revealed no change in expression of *cf60* and *ctnA*, which is consistent with previous findings of unaltered CtnA protein levels in *cln5* ^*-*^ cells (McLaren et al., 2021). Finally, upregulation of *smlA* (small aggregate formation protein) was observed, which encodes a protein that influences protein processing and secretion (Brock et al., 1996). Altogether, these observations support previous findings of Cln5 playing a role in aggregation and adhesion.

### 3.5 Loss of *cln5* affects the expression and activity of several carbohydrate enzymes

**FIGURE 6:**
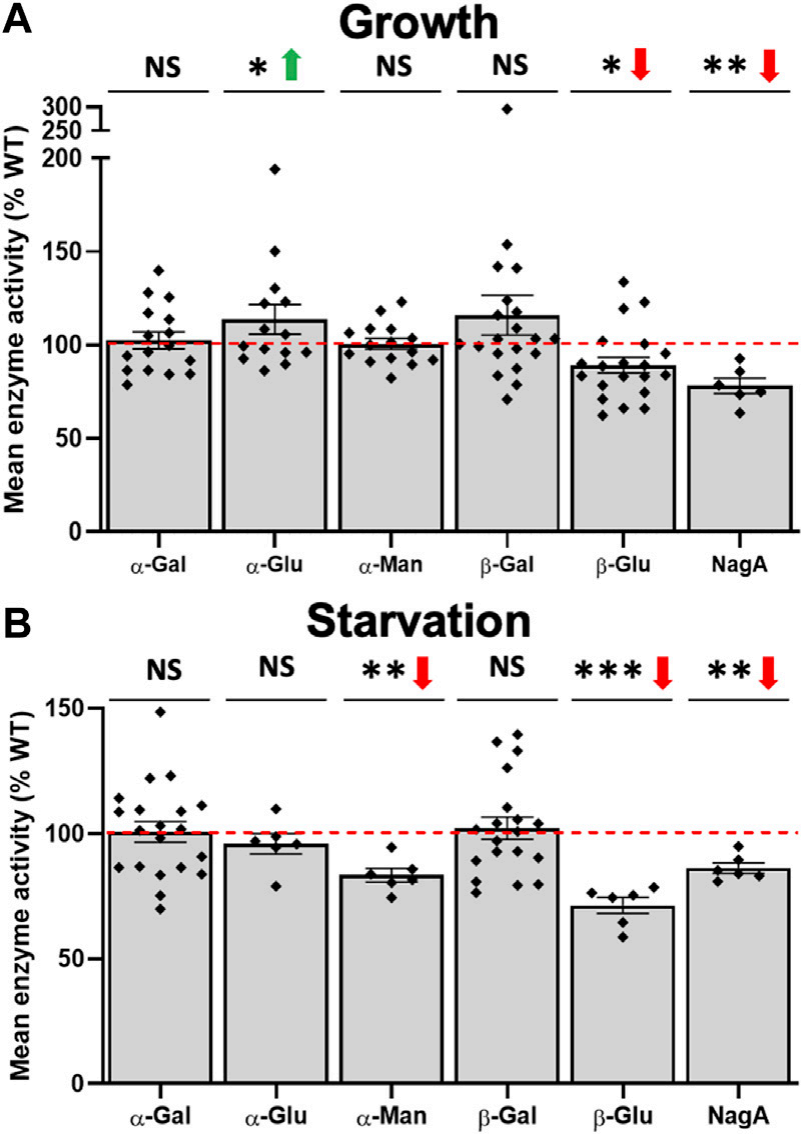
Loss of *cln5* affects the activity of carbohydrate enzymes during growth and starvation. WT and *cln5*-deficient cells during **(A)** growth and **(B)** 4-hour starvation were lysed and the activities of various carbohydrate enzymes were assessed including α-galactosidase (α-Gal), β-galactosidase (β-Gal), α-glucosidase (α-Glu), β-glucosidase (β-Glu), α-mannosidase (α-Man), and N-acetylglucosaminidase (NagA). Raw enzymatic values were subtracted from the blank solution and activities in *cln5*-deficient lysates were standardized against the activities in WT samples. Data presented as mean enzyme activity (% WT) ± SEM (*n* ≥ 6). *p < 0.05, **p < 0.01, ***p < 0.001 (one sample *t*-test). NS – not significant.

In previous studies, several lysosomal enzymes were identified as direct or indirect interactors of Cln5 including α-mannosidase, β-glucosidase, and NagA (Huber and Mathavarajah, 2018a). While the *D. discoideum* genome encodes several α-mannosidases, *manA* is the most highly expressed α-mannosidase gene during growth and aggregation (Stajdohar et al., 2017). The *D. discoideum* genome also encodes two β-galactosidases, *glb1* and *glb2*. Two α-galactosidases are encoded by the *D. discoideum* genome, *melA* and *DDB_G0291524*, with the latter being expressed more than *melA* during the *D. discoideum* life cycle. Two α-glucosidases (*gaa, modA*) are encoded by the *D. discoideum* genome with *modA* being expressed more than *gaa* during the life cycle (Stajdohar et al., 2017). Finally, the *D. discoideum* genome encodes one β-glucosidase (*gluA*). In our transcriptomics dataset, we observed many carbohydrate enzymes that were differentially expressed during *cln5* ^*-*^ growth and starvation. During growth, loss of *cln5* increased the expression of *glb1*, *manA*, *melA*, and *gaa* (Supplementary Table S8). When starved, *cln5* ^*-*^ cells increased the expression of *gluA* and decreased the expression of the putative α-galactosidase (*DDB_G0291524*) and α-mannosidase (*DDB_G0268754*) genes. Next, we assessed the intracellular activity of the aforementioned enzymes. During growth, loss of *cln5* decreased β-glucosidase and NagA activity and increased the activity of α-glucosidase (Figure 6A). During starvation, *cln5*-deficiency decreased α-mannosidase, β-glucosidase, and NagA activity (Figure 6B). We observed no effect of *cln5*-deficiency on α-galactosidase and β-galactosidase activity during both growth and starvation. Collectively, these findings suggest that Cln5 regulates the activities of several carbohydrate enzymes.

### 3.6 Loss of *cln5* affects the expression and activity of the proteasome

Ceroid lipofuscin accumulation is a common hallmark of the NCLs and has been postulated to occur due to alterations in the autophagy pathway (Cárcel-Trullols et al., 2015; Leinonen et al., 2017; Adams et al., 2019; Mukherjee et al., 2019; McLaren et al., 2021). Dysregulated autophagy has been observed in many CLN5 disease models including *D. discoideum* (Best et al., 2017; Leinonen et al., 2017; Adams et al., 2019; Doccini et al., 2020; McLaren et al., 2021). By scanning the list of DEGs, we identified genes involved in autophagy initiation (*atg1, atg13*) and autophagosome formation and turnover (*atg6A, atg8, atg18*) (all decreased) during growth, but no DEGs related to autophagy during starvation (Supplementary Table S9) (Tanida et al., 2005; Mizushima, 2010; Bento et al., 2016; Mesquita et al., 2017). Since our previous work in *D. discoideum* reported increased numbers of ubiquitin-positive proteins in *cln5* ^*-*^ cells during growth, we also examined our growth DEG list for genes associated with protein ubiquitination (Pickart, 2001; Jin et al., 2008; Williamson et al., 2009; Shao et al., 2013; McLaren et al., 2021). Not surprisingly, we identified DEGs that encode proteins involved in ubiquitin attachment, such as polyubiquitin proteins (*ubqA, ubqD, ubqF, ubqG, ubqH, ubqI, ubqJ*), all of which were downregulated during growth (Supplementary Table S10). In addition, DEGs encoding proteins involved in ubiquitin conjugation (E2 complex) were reduced, including ubiquitin-conjugating enzyme E2 (*ubcB*) and a gene similar to human *UBE2J2* (*ube2j2*), and genes encoding Ube2C and Ube2S (*ube2c* and *ube2s*, respectively) were elevated. Ubiquitin ligase genes (*skp1B, rnf160*) were also reduced in expression. However, some genes associated with the ligase activity of the E3 complex were increased such as subunits of the anaphase-promoting complex (*anapc3, anapc4, anapc5, anapc6, anapc7, anapc10, cdc20, cdc26*) (Supplementary Table S10) (Matyskiela et al., 2009; Wang et al., 2009). Finally, genes involved in protein deubiquitination such as ubiquitin hydrolases and thioesterases were reduced in expression (e.g., *uch2, yod1*) (Deol et al., 2020). Overall, these findings revealed the genetic impact of *cln5*-deficiency on the autophagy and protein ubiquitination pathways, which further supports the role of Cln5 in autophagy.

**FIGURE 7:**
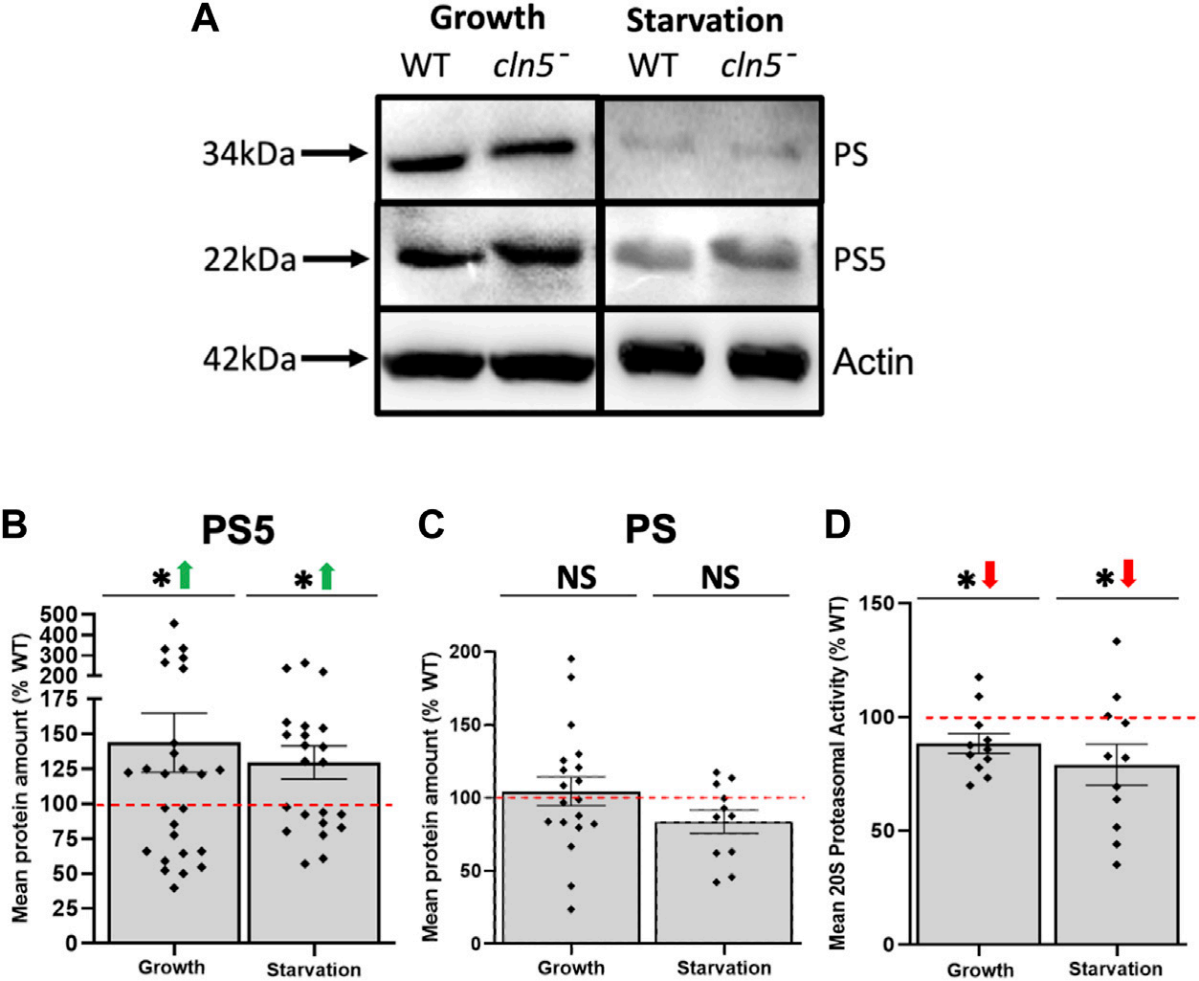
Loss of *cln5* increases the abundance of a proteasomal subunit during growth and starvation but decreases proteasome 20S activity. **(A)** Whole cell (WC) lysates from growth-phase and 4 h starved WT and *cln5* ^*-*^- cells were separated by SDS-PAGE and analyzed by western blotting. Membranes were probed with anti-PS, anti-PS5, and anti-β-actin (loading control). Fiji/ImageJ was used to quantify **(B)** PS5 and **(C)** PS protein bands, which were then standardized against the levels of β-actin. Data presented as mean protein amount (% WT) ± SEM (*n* ≥ 11). *p < 0.05 (one sample *t*-test). NS – not significant. **(D)** Proteasome 20S activity was assessed by collecting WT and *cln5* ^-^ cells during growth and after 4 h of starvation. Cells were then lysed and proteasome 20S activity was measured using a commercially available kit. Raw activity values were subtracted from the blank solution and activities in *cln5*-deficient lysates were standardized against the activities in WT samples. Data presented as mean proteasome 20S activity (% WT) ± SEM (n = 11). *p < 0.05 (one sample *t*-test).

Interestingly, the autophagy pathway and ubiquitin-proteasome system crosstalk with each other and both use ubiquitin as a tag to signal protein degradation (Kocaturk and Gozuacik, 2018). Our RNA-seq analysis showed that loss of *cln5* increased the expression of a gene predicted to be involved in proteasome assembly, *psmG4*, as well as reduced the expression of *psmE3*, which encodes a proteasome subunit that regulates the activity of the proteasome during *D. discoideum* growth (Supplementary Table S10) (Le Tallec et al., 2007; Masson et al., 2009). In addition, we observed elevated amounts of one proteasome subunit, PS5, during *cln5* ^-^ growth and starvation (Figures 7A,B), no effect on another proteasome subunit (Figure 7C), and decreased proteasome 20S activity during both growth and starvation (Figure 7D). These findings, coupled with previous work, suggest that Cln5 plays a role in proteasomal degradation and that the loss of *cln5* leads to dysfunctional protein turnover *via* both autophagy and through the proteasome.

### 3.7 Loss of *cln5* affects the expression and activity of other CLN-like proteins in *D. discoideum*

**FIGURE 8:**
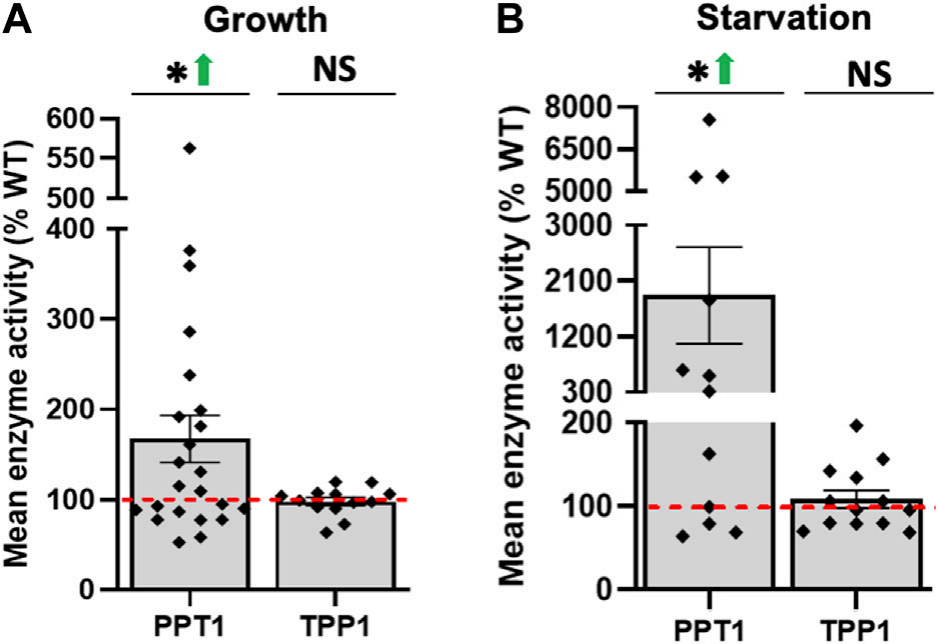
Loss of *cln5* increases PPT1 activity during growth and starvation but has no effect on TPP1 activity. **(A)** Growth-phase and **(B)** 4-hour starved WT and *cln5* ^-^ cells were lysed PPT1 and TPP1 activity in whole cell (WC) lysates were subtracted from the blank solution and activities in *cln5*-deficient lysates were standardized against the activities in WT samples. Data presented as mean enzyme activity (% WT) ± SEM (n ≥ 12). *p < 0.05 (one sample *t*-test). NS – not significant.

**FIGURE 9:**
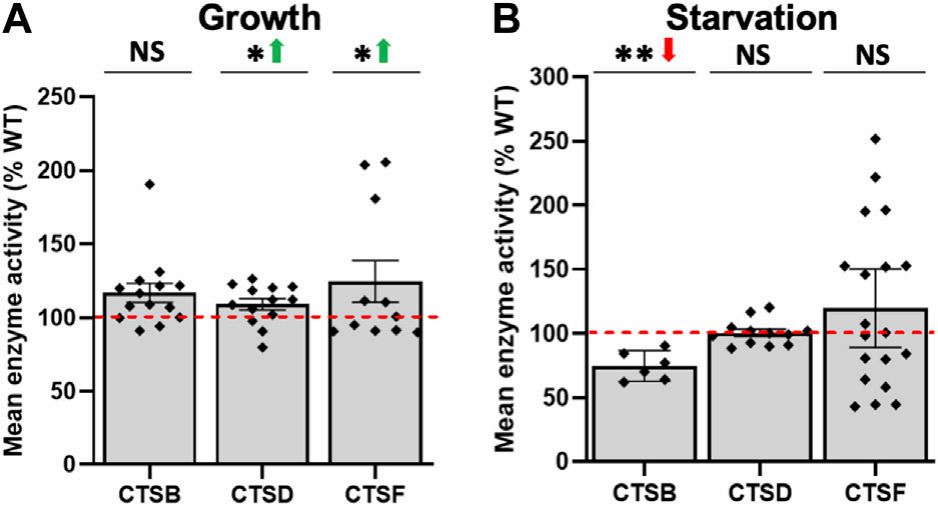
Loss of *cln5* affects the activity of cathepsins during growth and starvation. The activity of cathepsins were assessed during **(A)** growth and **(B)** 4-hour starvation in WT and *cln5* ^-^ cells as described in the Materials and methods. Activities in whole cell (WC) lysates were subtracted from the blank solution and activities in *cln5* ^*-*^ lysates were standardized against the activities in WT samples. Data presented as mean enzyme activity (% WT) ± SEM (n ≥ 6). *p < 0.05, **p < 0.01 (one sample *t*-test). NS – not significant.

It has been suggested that CLN proteins collectively participate in a shared biological pathway or pathways that converge to regulate a common cellular process (Persaud-Sawin et al., 2007; Huber, 2020). As a result, we scanned our list of DEGs and revealed several *CLN*-like genes that were differentially expressed in *cln5* ^*-*^ cells (Supplementary Table S11). During growth, *tpp1E* (similar to human *TPP1/CLN2*)*, ddj1* (similar to human *DNAJC5/CLN4*), and *kctd9* (similar to human *KCTD7/CLN14*) were reduced in expression in *cln5* ^*-*^ cells relative to WT cells, while *tpp1F* (similar to human *TPP1/CLN2*) and *grn* (similar to human *GRN/CLN11*) were elevated. During starvation, the expression of *tpp1B* and *tpp1F* (similar to human *TPP1/CLN2*)*, ctsD* (similar to human *CTSD/CLN10*), *DDB_G0291191* (similar to human *CTSF/CLN13*), and *DDB_G0269760* (similar to human *KCTD7/CLN14*) were increased, while *cprB* (similar to human *CTSF/CLN13*) was reduced. Based on these findings, we next assessed the intracellular activities of CLN proteins with demonstrated enzymatic activity in mammals including PPT1, TPP1, CTSD, and CTSF. While there was no effect of *cln5*-deficiency on *ppt1* expression, we detected increased PPT1 activity in both growth and starved cells (Figures 8A,B). Despite the effects of *cln5*-deficiency on *tpp1E* and *tpp1F* expression during growth, and *tpp1B* and *tpp1F* expression during starvation, there was no correlated impact on TPP1 activity in *cln5* ^*-*^ cells. There was elevated CTSD and CTSF activity observed in *cln5* ^*-*^ cells during growth but no changes were observed during starvation (Figures 9A,B). Finally, elevated *ctsB* expression was observed in *cln5* ^*-*^ cells during growth and cells starved for 4 h, but reduced CTSB activity was observed in *cln5* ^*-*^ cells during starvation. From these findings, it is evident that Cln5 influences the expression and activity of some CLN proteins, which further supports the molecular networking of *CLN* genes and proteins (Huber, 2020).

## 4 Discussion

In *D. discoideum*, *cln5*-deficiency suppresses cell proliferation, reduces cytokinesis, increases the basal level of autophagy, delays aggregation, impairs cAMP-mediated chemotaxis, reduces cell-substrate and cell-cell adhesion, and causes precocious multicellular development after mound formation (Huber and Mathavarajah, 2018b; McLaren et al., 2021). In this study, we used comparative transcriptomics to gain insight into the molecular mechanisms underlying these phenotypes. We then used the expression data to inform follow up work that examined the effect of *cln5*-deficiency on protein levels and enzyme activity during growth and the early stages of development.

Loss of *cln5* increases the intracellular levels of ubiquitinated proteins during growth (McLaren et al., 2021). Here, comparative transcriptomics revealed that the most significantly downregulated genes during growth were those that encode heat shock proteins and proteins associated with protein ubiquitination, which could reflect an attempt by *cln5* ^*-*^ cells to limit the expression of genes linked to protein ubiquitination. The most significantly upregulated genes were associated with cell cycle progression and mitosis. Since loss of *cln5* inhibits cell proliferation (McLaren et al., 2021), these findings indicate that *cln5* ^*-*^ cells increase the expression of genes linked to cell proliferation to help restore the normal rate of proliferation. During starvation, the most significantly downregulated genes were linked to development. Conversely, the most significantly upregulated genes were involved in intracellular trafficking and cell-cell adhesion. These findings are consistent with the defects in developmental timing and adhesion observed in *cln5* ^*-*^ cells (Huber and Mathavarajah, 2018b; McLaren et al., 2021).

GO term analysis revealed an enrichment of DEGs associated with autophagy, protein homeostasis, and lipid metabolism during growth. These findings are consistent with work in *D. discoideum* and mammalian models of CLN5 disease that have linked the function of CLN5 to these cellular processes (El Haddad et al., 2012; Schmiedt et al., 2012; Best et al., 2017; Leinonen et al., 2017; Adams et al., 2019; Doccini et al., 2020; McLaren et al., 2021; Yasa et al., 2021; Doccini et al., 2022). Previous work suggests that CLN5 functions as either a glycoside hydrolase or depalmitoylase (Huber and Mathavarajah, 2018a; Luebben et al., 2022). Here, comparative transcriptomics showed that loss of *cln5* during growth alters the expression of genes that encode enzymes. During starvation, GO term analysis identified an enrichment of DEGs that encode enzymes and proteins involved in catabolic processes. In addition, consistent with the extracellular localization of Cln5 in *D. discoideum* and CLN5 in mammals (Isosomppi et al., 2002; Moharir et al., 2013; Hughes et al., 2014; Huber and Mathavarajah, 2018a; Huber and Mathavarajah, 2018b; McLaren et al., 2021), GO term analyses identified an enrichment of DEGs that encode proteins that localize to secretory vesicles, the cell periphery, the plasma membrane, and extracellularly, which is consistent with previous work that indicated a role for CLN5 in protein secretion (Hersrud et al., 2016; Huber, 2021; Iwan et al., 2021). Notably, GO term enrichment analyses also showed that loss of *cln5* affects the expression of genes that encode lysosomal enzymes, which aligns with the lysosomal localization of CLN5 in mammalian cells (Isosomppi et al., 2002).

LAGO analysis revealed altered expression of genes linked to cell cycle progression and mitosis in *cln5* ^*-*^ cells, which is consistent with the reduced proliferation of *cln5* ^*-*^ cells (McLaren et al., 2021). In addition, we showed that loss of *cln5* significantly increases the expression of *aprA*, which encodes the well-established proliferation repressor AprA (Brock and Gomer, 2005). Intriguingly, *cln5*-deficiency decreased the intracellular amount of AprA, but increased the extracellular amount, suggesting that loss of *cln5* increases AprA secretion. These findings support the reduced proliferation of *cln5* ^*-*^ cells since more AprA is present outside cells to repress proliferation. In addition, it appears that *cln5* ^*-*^ cells increase the expression of *aprA* to counteract the reduced intracellular amount of the protein. Finally, loss of *cln5* also increased the intracellular and extracellular levels of CfaD, which participates in AprA-dependent signalling. Combined, these observations indicate that *cln5* ^*-*^ cells cannot modulate AprA-dependent signalling and the increased secretion of AprA is exacerbated by the increased expression of *aprA* in *cln5*-deficient cells, which ultimately supresses cell proliferation.

Our previous work in *D. discoideum* reported reduced cytokinesis in *cln5*-deficient cells (McLaren et al., 2021). Here, RNA-seq revealed reduced expression of genes associated with cytokinesis including *ctxA*, *mhcA*, and *vinA*. We also observed significantly increased amounts of MhcA protein in *cln5* ^*-*^ cells, which could explain the reduced expression of *mhcA*, and reflect an attempt by *cln5* ^*-*^ cells to mitigate defects in MhcA-dependent cytokinesis. Interestingly, our RNA-seq analysis revealed a cluster of DEGs that encode proteins that bind to the cytoskeleton, which is consistent with a previous study that revealed DEGs in *Cln1* ^*−/−*^ mice related to cytoskeleton organization (von Schantz et al., 2008). Moreover, altered cytokinesis has also been reported in yeast and *D. discoideum* knockout models of CLN3 disease (Codlin et al., 2008; Mathavarajah et al., 2018) indicating that multiple *CLN* genes regulate cytokinesis.

Comparative transcriptomics identified DEGs associated with aggregation, cAMP-mediated chemotaxis, and cell adhesion in *cln5* ^*-*^ cells, which is consistent with the delayed aggregation, suppressed cAMP-mediated chemotaxis, and reduced adhesion observed in *cln5*-deficient cells (Huber and Mathavarajah, 2018b; McLaren et al., 2021). We also found that loss of *cln5* elevates the amount of secreted CmfA and expression of the gene encoding the CmfA receptor, *cmfB*, which together modulate cAMP-mediated chemotaxis and the expression of early developmental genes (Gomer et al., 1991; Yuen et al., 1995; van Haastert et al., 1996; Deery and Gomer, 1999). In addition, *cln5*-deficiency increased the expression of the gene encoding the cAMP phosphodiesterase RegA. These findings suggest that the delayed aggregation of *cln5* ^*-*^ cells can be at least partly due to altered CmfA-dependent signalling and increased degradation of cAMP due to elevated expression of *regA*. Finally, consistent with the role of Cln5 in cell adhesion (Huber and Mathavarajah, 2018b), loss of *cln5* increased the expression of genes that encode subunits of discoidin, which resulted in a correlated increase in the intracellular and extracellular amounts of discoidin protein in starved cells. Discoidins are secreted lectins that regulate cell migration and cell-substrate adhesion in *D. discoideum* (Springer et al., 1984; Barondes et al., 1985). Thus, the increased expression and amount of discoidin protein likely reflected an attempt by *cln5*-deficient cells to restore adhesion to WT levels.

RNA-seq identified DEGs in *cln5* ^*-*^ cells associated with protein tagging, protein degradation, and autophagy, which aligns with studies from *D. discoideum*, mice, and humans linking the function of CLN5 to autophagy (Best et al., 2017; Leinonen et al., 2017; Huber and Mathavarajah, 2018b; Adams et al., 2019; Doccini et al., 2020; Basak et al., 2021b; McLaren et al., 2021). Intriguingly, autophagy is dysregulated in many NCL subtypes (Kim et al., 2022). Accumulated evidence suggests that autophagy and the ubiquitin-proteasome system work together to regulate protein degradation and operate in a compensatory manner when one of the pathways is inhibited (Kocaturk and Gozuacik, 2018). Our previous work showed that loss of *cln5* increases the basal level of autophagy during growth (McLaren et al., 2021). Here, we reported an accumulation of one of the proteasomal subunits in *cln5* ^*-*^ cells, but a reduction in proteasome 20S activity during both growth and starvation, which is consistent with the increased abundance of ubiquitin-positive proteins in *cln5* ^*-*^ cells (McLaren et al., 2021). Together, these results indicate that Cln5 influences protein degradation *via* autophagy and the ubiquitin-proteasome system.

Autophagy is dependent on lysosomal function. Not surprisingly, our RNA-seq dataset contained several DEGs in *cln5* ^*-*^ cells that encode carbohydrate enzymes including β-glucosidase and α-mannosidase, which both interact with Cln5 (Huber and Mathavarajah, 2018a). We followed up this analysis by showing that *cln5*-deficiency affects the activity of various carbohydrate enzymes. Notably, *cln5* ^*-*^ cells displayed reduced NagA activity during growth and starvation, which is consistent with previous work that reported that *D. discoideum* Cln5 and human CLN5 can also cleave the NagA substrate and that Cln5 and NagA may participate in a common pathway in *D. discoideum* that regulates autophagy (Huber and Mathavarajah, 2018a; McLaren et al., 2021). We also showed that loss of *cln5* alters the expression and activities of other CLN-like proteins in *D. discoideum*. This aligns with previous work that reported an interaction between CLN5 and PPT1 in COS-1 cells (Lyly et al., 2009). In addition, *D. discoideum* Cln5 interacts with CtsD, various homologs of human CTSF (CprA, CprD, CprE, and CprG), NagA, α-mannosidase, and β-glucosidase, all of which may have displayed altered enzymatic activity due to the absence, and hence, lack of interaction with Cln5, in *cln5* ^*-*^ cells (Huber and Mathavarajah, 2018a). While our previous work showed that loss of *cln5* decreases the intracellular amount of CtsD during starvation (McLaren et al., 2021), in this study, comparative transcriptomics revealed increased expression of *ctsD* in starved *cln5* ^*-*^ cells. These findings indicate that *cln5* ^*-*^ cells upregulate the expression of *ctsD* to restore the intracellular amount of the protein. Also, our work showed that loss of *cln5* elevates *ctsB* expression but reduces CTSB activity. Previous work also reported reduced CTSB activity in *CLN5*-deficient cells and it was suggested that the loss of *CLN5* impairs intracellular trafficking and the movement of lysosomes (Mamo et al., 2012; Basak et al., 2021a; Yasa et al., 2021). Consistent with these findings, LAGO analyses revealed an enrichment of GO terms associated with intracellular vesicles including lysosomes, endocytic vesicles, and phagocytic vesicles. In CLN5-depleted HeLa cells, endosomal sorting is perturbed due to abolished retromer interaction and recruitment to the endosome, as well as poor fusion between the lysosome and autophagosome (Mamo et al., 2012; Yasa et al., 2021). Combined, these findings indicate that loss or mutation of *CLN5* affects the expression, synthesis, and/or delivery of enzymes to lysosomes.

In conclusion, this study sheds light on the pathways perturbed in *cln5* ^*-*^ cells, highlights the multifaceted role of CLN5 in eukaryotic cells, and contributes knowledge to the mechanisms that may be disrupted in CLN5 disease patients.

## Data Availability

The original contributions presented in the study are publicly available. This data can be found here: https://www.ncbi.nlm.nih.gov/bioproject/?term=PRJNA888040.

## Glossary

AprA: autocrine proliferation repressor
BH: Benjamin-Hochberg
CadA: calcium-dependent cell adhesion molecule
cAMP: cyclic adenosine monophosphate
carD: cAMP receptor D
CB: conditioned buffer
CfaD: counting factor-associated protein D
CLN: ceroid lipofuscinosis neuronal
CM: conditioned media
CmfA: conditioned medium factor
CmfB: conditioned medium factor receptor
CtnA: countin
CTSB: cathepsin B
CTSD: cathepsin D
CTSF: cathepsin F
CtxA: cortexillin I
CtxB: cortexillin II
DEG: differentially expressed gene
Dsc: discoidin
GO: gene ontology
HISAT2: hierarchical indexing for spliced alignment of transcripts 2
LAGO: logically accelerated gene ontology
MES: 2-(N-morpholino)ethanesulfonic acid
MhcA: myosin heavy chain A type II
NagA: N-acetylglucosaminidase A
NCL: neuronal ceroid lipofuscinosis
PPT1: palmitoyl-protein thioesterase 1
PS: proteasomal subunit
RegA: 3′,5′-cyclic-nucleotide phosphodiesterase
RNA-seq: RNA sequencing
SARTools: statistical analysis of RNA-seq data tools
TPP1: tripeptidyl peptidase 1
VinA: vinculin A
WC: whole cell
WT: wild-type
